# Pharmacokinetic and Pharmacodynamic Assessments of the Ivermectin and Levamisole Combination to Control Resistant Nematodes in Cattle

**DOI:** 10.3390/pharmaceutics18050630

**Published:** 2026-05-21

**Authors:** Candela Canton, Laura Ceballos, Lucila Canton, Laura Moreno, Paula Domínguez, Luis Alvarez, Carlos Lanusse

**Affiliations:** Laboratorio de Farmacología, Centro de Investigación Veterinaria de Tandil (CIVETAN), CONICET, Facultad de Ciencias Veterinarias, UNCPBA, Campus Universitario, Tandil 7000, Argentina; lauceballosf@gmail.com (L.C.); cantonlu@vet.unicen.edu.ar (L.C.); lmoreno@vet.unicen.edu.ar (L.M.); pauladom@vet.unicen.edu.ar (P.D.); lalvarez@vet.unicen.edu.ar (L.A.); clanusse@vet.unicen.edu.ar (C.L.)

**Keywords:** ivermectin, levamisole, anthelmintic combination, pharmacokinetic, pharmacodynamic assessment, resistant nematodes

## Abstract

**Background/Objectives:** Combination of antiparasitic drugs with different mechanisms of action has been suggested as an effective strategy to delay the development of parasite resistance. Considering the need to understand the pharmacological basis of drug combinations, the current study evaluated the potential pharmacokinetic (PK) interactions and the clinical efficacy (pharmacodynamic response) occurring after the subcutaneous administration of ivermectin (IVM) and levamisole (LEV), administered either as single treatments or concurrently to different groups of parasitized calves on three commercial farms (A, B and C). **Methods:** Forty-five (45) male calves naturally infected with gastrointestinal nematodes were randomly allocated into three groups (*n* = 15): IVM, treated with IVM by subcutaneous injection (0.2 mg/kg); LEV, treated subcutaneously with LEV (8 mg/kg); IVM + LEV, simultaneously treated with IVM and LEV (two subcutaneous injections at the same dose rates). Seven animals from each treated group (farm C) were randomly selected to perform the PK study. Drug concentrations were measured by HPLC. The therapeutic response (efficacy) was determined at 14 days after treatment by the fecal egg reduction test. **Results:** The mean area under the concentration vs time curve (AUC) for IVM obtained after administration of IVM alone (274 ± 65.1 ng.d/mL) was similar to that obtained when IVM was co-administered with LEV (295 ± 111 ng.d/mL). Likewise, mean LEV AUC values were similar after LEV administration alone (8.90 ± 2.69 µg.h/mL) or combined with IVM (9.11 ± 1.82 µg.h/mL). No adverse PK interactions were observed after the combined treatment, with similar PK parameters (*p* > 0.05) obtained between the single-drug and the combination-based strategies. On farm A, the overall fecal egg reductions were 38% (IVM), 99% (LEV) and 100% (IVM + LEV). While *Cooperia* spp. and *Haemonchus* spp. showed reduced susceptibility to IVM treatment, LEV demonstrated high efficacy against both genera, with only a minimal proportion of *Haemonchus* spp. remaining after treatment. Similarly, total fecal egg reductions were 42% (IVM), 99% (LEV) and 100% (IVM + LEV) on farm B, and 54% (IVM), 99% (LEV) and 100% (IVM + LEV) on farm C. On those farms, IVM was ineffective against *Cooperia* spp. and/or *Haemonchus* spp., while LEV failed to control *Ostertagia* spp. Remarkably, the combination of both molecules was the only treatment that achieved 100% efficacy against all nematode genera (*Cooperia*, *Ostertagia*, *Haemonchus* and *Oesophagostomum* spp.). **Conclusions:** Based on the described PK and pharmacodynamic (PD) assessments, the IVM + LEV combination appears to be a promising pharmacological option for controlling resistant gastrointestinal nematodes in cattle, with the additional potential to delay the progression of nematode anthelmintic resistance. Overall, the study provides original and robust pharmacokinetic and efficacy data that contribute to the optimization of parasite control strategies in cattle. This drug combination strategy may enhance treatment efficacy and contribute to improved parasite control in cattle production systems.

## 1. Introduction

Drug resistance in gastrointestinal (GI) nematodes of livestock has escalated globally and is now considered one of the major sanitary and productivity constraints in ruminant production systems [1,2]. Despite the urgent need for new antiparasitic chemical classes with novel molecular targets, drug discovery and development have progressed slowly [3]. Consequently, optimizing the pharmacological use of existing compounds has become a research priority and a central strategy to mitigate resistance development. In this scenario, knowledge of drug pharmacokinetics (PK) and pharmacodynamics (PD) is critical to design rational parasite control programs for livestock animals [4,5].

The reduced therapeutic response of single-drug treatments has accelerated the search for combination therapies as a resistance-management strategy. The use of anthelmintic drugs from different chemical classes can help delay the development of resistance by reducing the likelihood that parasites resistant to one compound will survive treatment [6,7]. The theoretical basis for drug combinations is grounded in population genetics principles: when two actives with independent modes of action are administered simultaneously at fully effective doses, parasites resistant to one compound are expected to be removed by the second. Consequently, only worms carrying resistance alleles to both drugs would be expected to survive, and such multi-resistant genotypes are likely to occur at lower frequencies and may be associated with fitness costs [8].

**Figure 1:**
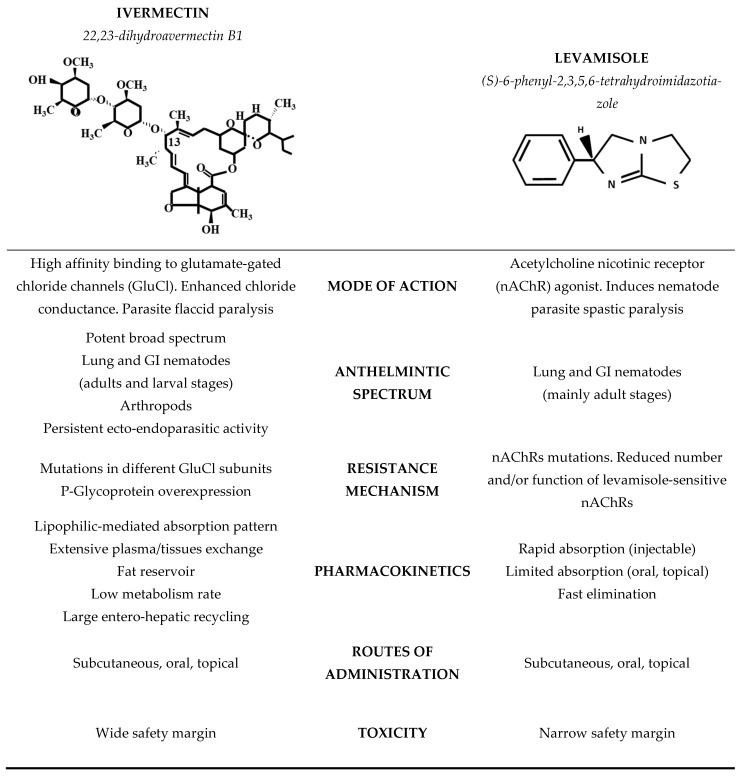
Chemical structures and main pharmacological features of the two (2) active principles used as a combination to improve parasite control: ivermectin (a potent broad spectrum 16-membered macrocyclic lactone ecto-endo antiparasitic drug) [12,13,19,20] and levamisole (a narrow spectrum antinematodal drug from the imidazothiazoles family) [16,21,22,23]. The information presented in this figure was adapted from Riviere [10].

Ivermectin (IVM) and levamisole (LEV) are two anthelmintics that differ markedly both in their pharmacological properties and in their mechanisms of action (see Figure 1). IVM is a potent broad-spectrum antiparasitic drug, extensively used in veterinary medicine [9]. It is a highly lipophilic compound belonging to the macrocyclic lactone family of avermectins and is one of the most widely used endectocides in livestock animals [10]. It is highly effective against adults as well as the developing and hypobiotic larvae of most GI nematodes, lungworms [11] and many arthropods ectoparasites in cattle [12]. After subcutaneous (SC) administration in cattle, its low aqueous solubility results in slow absorption from the injection site, which contributes to prolonged systemic availability [13]. IVM is extensively distributed to peripheral tissues, including GI mucosa, lungs, and skin [14], where tissue concentrations exceed plasma concentration and persist for extended periods, supporting sustained antiparasitic activity. From a mechanistic standpoint, IVM binds with high affinity to glutamate-gated chloride channels in nerve and muscle cells, increasing membrane permeability to chloride ions and inducing flaccid paralysis and death [12,15].

Conversely, LEV is the main imidazothiazole anthelmintic used in veterinary medicine. It is a narrow-spectrum nematodicidal drug approved for use in several animal species, being active against GI and pulmonary nematode parasites, but it has no efficacy against cestodes or trematodes [16]. In comparison with IVM, LEV is rapidly absorbed after parenteral administration, reaching peak plasma concentrations within 0.5–2 h in cattle, is widely distributed, and is rapidly eliminated, with short elimination half-lives (approximately 4–6 h) [17,18].

With regard to its mechanism of action, and in clear contrast to IVM, LEV induces spastic paralysis by selectively activating nicotinic acetylcholine receptor ion channels located on nematode nerve and muscle cells [21]. Overall, LEV is characterized by rapid neuromuscular action, short systemic persistence, and broad efficacy against adult nematodes. The different molecular targets of IVM and LEV provide the rationale for evaluating their concurrent administration in cattle in the present study [6].

However, when two molecules are co-administered in vivo, potential PK interactions (e.g., interferences on absorption, systemic exposure, tissue distribution, elimination rates) or PD interactions (additive, synergistic, or antagonistic effects) must be considered. Given that systemic exposure is a key determinant of drug efficacy against target parasites, especially in cattle, where formulation and route of administration influence drug disposition [4], PK and PD evaluations under field conditions are essential before recommending the practical use of combined treatments. Therefore, pharmaco-parasitological studies assessing both drug exposure and clinical responses are required to determine whether the IVM + LEV combination provides optimized efficacy while contributing to resistance mitigation in cattle. Considering these aspects, the combined use of IVM and LEV to improve the treatment of GI helminth infections was investigated in the present study.

The current study evaluated the therapeutic response and the potential PK interactions occurring after the SC administration of IVM and LEV, either as single treatments or concurrently, to calves naturally infected with resistant GI nematodes on three commercial cattle farms.

## 2. Materials and Methods

### 2.1. Field Trial and Animals

The study was conducted on three commercial cattle farms located in the Humid Pampean Region of Argentina. In these farms, as in most cattle production systems in the country, beef production is based on grazing. The resistance status of GI nematode populations on each farm had been previously determined using the fecal egg count reduction test (FECRT) [24]. All selected farms (A, B, and C) showed a predominance of IVM-resistant nematode populations.

Forty-five (45) male Aberdeen Angus calves, aged 9–12 months old and naturally infected with GI nematodes resistant to macrocyclic lactones, were recruited in this trial. On day −1, all the animals were checked for worm egg per gram (EPG) counts and ear tagged, and the individual body weights were recorded. Animals with at least 100 EPG on day −1 were selected for inclusion in the study.

Animal procedures and management protocols were approved by the Ethics Committee (act 11/2020) of the Facultad de Cs. Veterinarias, Universidad Nacional del Centro de la Provincia de Buenos Aires (UNCPBA), Tandil, Argentina.

### 2.2. Experimental Design and Treatments

Selected animals were blocked by pre-treatment EPG counts and randomly assigned into groups of 15 animals each. The experimental groups were: IVM, animals were treated with IVM (Ivomec^®^, a propylene glycol 60%/glycerol formal 40% (*v*/*v*) 1% solution, Boehringer Ingelheim, Munro, Argentina) by the SC route at 0.2 mg/kg; LEV, animals were treated with LEV (Ripercol^®^, an 18.8% levamisole phosphate solution, Zoetis, Buenos Aires, Argentina) by the SC route at 8 mg/kg; and IVM + LEV, animals were treated with both IVM and LEV (separate injections) administered at 0.2 and 8 mg/kg, respectively. Adverse events were assessed based on clinical observations throughout the 24 h after each anthelmintic treatment.

### 2.3. PK Study

The PK study was carried out on farm C. Seven randomly selected animals from each treated group were used in the PK study. Blood samples (10 mL) were taken from the jugular vein in heparinised Vacutainer^®^ tubes (Becton Dickinson, Franklin Lakes, NJ, USA) as follows: IVM and IVM + LEV groups, before treatment and at 2, 4, 6, 8 and 12 h and 1, 3, 5, 7, 15, 20 and 30 days post-treatment; LEV and IVM + LEV, before treatment and at 1, 2, 4, 6, 8, 10, 12, 16, 20, 24 and 28 h post-treatment. Animals in the IVM + LEV group were sampled once, following a combined sampling schedule that included all time points required for the determination of both compounds. Plasma was separated by centrifugation at 3000× *g* for 15 min, placed into plastic tubes and frozen at −20 °C until analysis by High Performance Liquid Chromatography (HPLC).

### 2.4. Analytical Procedures

#### 2.4.1. IVM Analysis

The extraction of IVM from spiked and experimental plasma samples was carried out following an adaptation of the technique described by Lifschitz [25]. An aliquot of 0.25 mL of plasma sample was combined with doramectin (DRM) (used as internal standard) and then 1 mL of acetonitrile (J.T. Baker, Phillipsburg, NJ, USA) was added to each sample. After mixing for 20 min, samples were sonicated in an ultrasonic bath for 10 min (Transonic 570/H, Laboratory Line Instruments Inc., Melrose Park, IL, USA). The solvent–sample mixture was centrifuged at 2000× *g* for 15 min and the supernatant was manually transferred into a tube and concentrated to dryness under a stream of nitrogen gas. The resuspension was carried out with a solution of N-methylimidazole (Sigma Chemical, St. Louis, MO, USA) in acetonitrile (1:1) [26]. Derivatization was initiated by adding trifluoroacetic anhydride (Sigma Chemical, St Louis, MO, USA) solution in acetonitrile (1:2). Finally, an aliquot of this solution was injected directly into the chromatographic system. IVM concentrations were determined by HPLC using a Shimadzu 10 A-HPLC system with a fluorescence detector (Shimadzu, RF-10 Spectrofluorometric detector, Kyoto, Japan). The HPLC methodology for IVM quantification was validated. Calibration curves were prepared in the range between 0.2 and 100 ng/mL. The statistical program Instat 3.0 (Graph Pad Software Inc., San Diego, CA, USA) was used for linear regression analyses and linearity tests. Linearity was established to determine the relationship between IVM concentrations and the corresponding analyte-to-internal standard response ratio. The relative recovery, precision and limit of quantification were also defined. The linear regression lines for IVM showed correlation coefficients ≥ 0.99. The mean recovery percentage for concentrations ranging between 0.2 and 100 ng/mL (*n* = 6) was 88% with CV of 10.5%. The inter-day and intra-day precisions of the extraction and chromatography procedures were estimated by processing replicate aliquots (*n* = 4) of samples containing known IVM concentrations. The precision of the analytical procedures obtained after HPLC analysis showed a CV < 6%. The LOQ was established at 0.2 ng/mL.

#### 2.4.2. LEV Analysis

The extraction of LEV from spiked and experimental plasma samples was carried out following an adaptation of the technique described by Canton [17]. Plasma samples (1000 µL) were placed into C18 SPE cartridges (Strata^®^, 100 mg, Phenomenex, CA, USA) previously conditioned. They were sequentially washed with 1 mL of water, eluted with 1.5 mL of HPLC-grade methanol, and concentrated to dryness under a stream of nitrogen at 56 °C in a water bath. The dried residue was reconstituted with 250 µL of mobile phase, a phosphoric acid 85% in triethylamine/methanol/acetonitrile/water (0.32/0.5/15.5/83.36). Finally, 100 µL of this solution was injected into the chromatographic system. LEV concentrations were determined by HPLC using a Shimadzu HPLC system with autosampler (Shimadzu Corporation, Kyoto, Japan). HPLC analysis was undertaken using a C18 column (Phenomenex, 5 µm, 4.6 mm × 250 mm) at a flow rate of 1.2 mL/min. There was no interference of endogenous compounds in the chromatographic determinations. The HPLC methodology for LEV quantification was validated. Calibration curves were prepared in the range between 0.10 and 2 µg/mL. The statistical program Instat 3.0 (Graph Pad Software Inc., San Diego, CA, USA) was used for linear regression analyses and linearity tests. Linearity was established to determine the LEV concentrations/detector responses relationship. The absolute recovery, precision and limit of quantification were also defined. The linear regression lines for LEV showed correlation coefficients ≥ 0.99. The mean recovery percentage for concentrations ranging between 0.10 and 2 µg/mL (*n* = 6) was 80% with CV of 10.3%. The inter-day and intra-day precisions of the extraction and chromatography procedures were estimated by processing replicate aliquots (*n* = 4) of samples containing known LEV concentrations. The precision of the analytical procedures obtained after HPLC analysis showed a CV < 10%. The LOQ was established at 0.10 µg/mL.

### 2.5. Pharmacokinetic Analysis of the Data

Data concentration profiles for each analyte, obtained after the treatment of each individual animal, were analyzed using a non-compartmental approach with version 2.0 of the PkSolutions software (Summit Research Service, Montrose, CO, USA). The peak concentration (Cmax) and time to peak concentration (Tmax) were recorded directly from the measured concentration data. Pharmacokinetic parameters were determined. The elimination half-life (T_½el_) was calculated as ln2/λ_el_, where λ_el_ is the slope of the terminal phase. The rates were calculated by performing regression analysis using data points belonging to the terminal phase concentration–time plot. The area under the plasma concentration–time curve from zero up to the quantification limit (AUC_0–t_) was calculated using the trapezoidal rule [27] and further extrapolated to infinity (AUC_0–∞_) by dividing the last experimental concentration by the terminal elimination rate constant. Statistical moment theory was applied to calculate the mean residence time (MRT) according to Perrier [28].

### 2.6. Anthelmintic Efficacy Trial: Fecal Egg Count Reduction Test and Coprocultures

Fecal samples were individually collected directly from the rectum of each calf during pre-treatment (day −1) and again on day 14 post-treatment. EPG counts were performed by a modified McMaster technique with a sensitivity of 10 EPG [29]. Additionally, 10 g of feces (obtained from an individual animal and/or from a pool of animals from each experimental group) were used to prepare coprocultures on each sampling day. The nematode genera were identified through the third-stage larvae (L3) recovered from these coprocultures [30]. L3 were collected by the Baermann technique and approximately 100 L3 were differentiated from each sample. Thus, the relative participation of each genus per experimental group was determined.

The anthelmintic efficacy of the different treatments was assessed by the FECRT, according to the recommendations of the last WAAVP guidelines [31]. The data analysis was conducted using the FECRT web-based platform (www.fecrt.com accessed on 7 November 2025), applying the delta method as described by Levecke [32].

In addition, efficacy against different genera was calculated via partitioning the mean fecal egg count of each treatment group pre- and post-treatment by the proportion of L3 of each genus in the corresponding coproculture [33].

### 2.7. Statistical Analysis of the Data

The PK parameters and concentration data are reported as arithmetic mean ± Standard Deviation (SD). PK parameters for IVM and LEV, calculated after the single or combined administration of IVM and LEV, were statistically compared using Student’s *t*-test or ANOVA + Tukey. Fecal egg counts (reported as arithmetic mean ± SD) were compared by non-parametric Kruskal–Wallis test. A value of *p* < 0.05 was considered statistically significant. The statistical analysis was performed using the Instat 3.0 software (Graph Pad Software, San Diego, CA, USA).

## 3. Results

### 3.1. Pharmacokinetic Study

**Figure 2:**
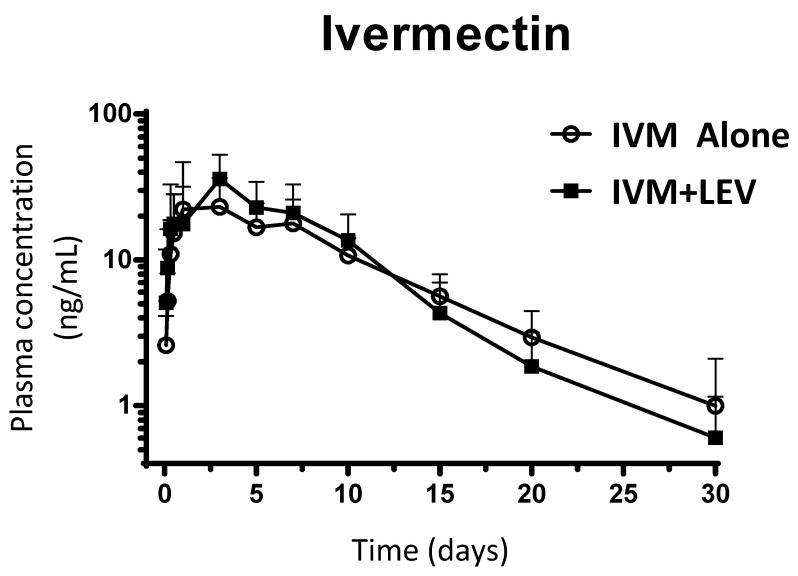
Mean (± SD) plasma concentration–time profiles of ivermectin (IVM) following subcutaneous administration either as a single treatment (0.2 mg/kg) or co-administered with levamisole (LEV, 8 mg/kg) in parasitized calves (*n* = 7).

IVM was the main analyte recovered in plasma after SC administration of IVM to beef cattle. The mean (± SD) plasma concentration profiles of IVM after its administration both alone and co-administered with LEV are shown in Figure 2. IVM plasma concentrations were measured up to 30 days post-treatment. Table 1 summarizes the main PK parameters for IVM obtained after the administration of IVM to beef cattle either alone or co-administered with LEV. No statistical differences between single- and combined-based treatments were observed (*p* > 0.05) for all experimental groups. Therefore, the presence of LEV did not affect the plasma disposition kinetics of IVM after the combined treatment. A limitation of the present study is the potentially limited statistical power associated with the sample size used, although this sample size is consistent with those commonly employed in pharmacokinetic studies in cattle.

**Figure 3:**
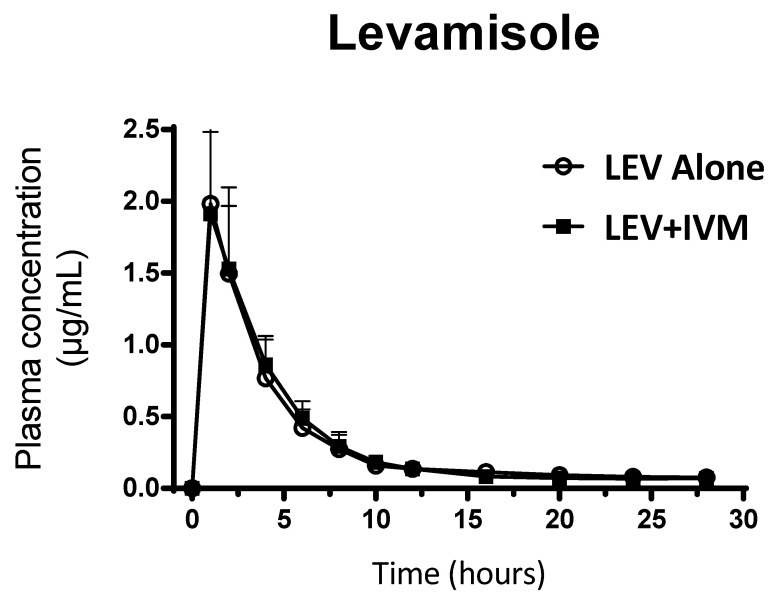
Mean (± SD) plasma concentration–time profiles of levamisole (LEV) following subcutaneous administration either as a single treatment (8 mg/kg) or co-administered with ivermectin (IVM, 0.2 mg/kg) in parasitized calves (*n* = 7).

LEV was the main analyte recovered in plasma after SC administration of LEV. Figure 3 shows the mean (± SD) plasma concentrations profiles of LEV after its SC administration both alone and co-administered with IVM. This compound was detected in plasma between 1 h and 28 h post-treatment. No statistical differences between both treatments were observed (*p* > 0.05). Therefore, the plasma disposition kinetics for LEV did not show differences between the single-drug and the combined-based treatment. Table 2 summarizes the plasma PK parameters for LEV both alone and co-administered with IVM.

### 3.2. Anthelmintic Efficacy Trial

All anthelmintic treatments were well tolerated as no adverse events were observed in treated animals. Experimental animals had an average of 697 EPG counts ranging from 280 to 1400 on farm A, 537 EPG counts ranging from 100 to 1820 on farm B, and 337 EPG counts ranging from 200 to 1140 on farm C. The mean EPG counts were similar (*p* > 0.05) across all groups on each farm at the beginning of the trial. Table 3 presents the overall fecal egg counts recorded across all farms on day 14 post-treatment, along with the lower and upper 90% confidence intervals (CI) and the corresponding nematode population status. The analysis of the 90% CI confirmed the presence of IVM-resistant nematodes on all the farms included in the study. In fact, the 90% CI ranged from 3.3% to 83% across all farms. In contrast, regarding LEV, the 90% CI ranged between 96.8% and 100%, indicating that the nematode population on the three farms were susceptible to this anthelmintic. The mean EPG counts were not statistically different between the treatment groups on Day −1 (*p* > 0.05) but differed on Day 14 (*p* < 0.05) on all farms. In this context, the EPG counts after LEV alone and co-administered with IVM were significantly (*p* < 0.05) lower than the egg counts after IVM. Although no significant differences in post-treatment EPG counts were found between LEV alone and IVM + LEV, the combined treatment was the only one that reached 100% anthelmintic efficacy.

The anthelmintic efficacies against *Cooperia* spp., *Haemonchus* spp., *Ostertagia* spp. and *Oesophagostomum* spp. for the different treatments are shown in Table 4. On farms A and B, IVM failed to control *Haemonchus* spp. and *Cooperia* spp., showing efficacies ranging from 8.7% to 87%. On farm C, *Cooperia* spp. was the only genus resistant to IVM (FECR 40.9%). While, on farm A, some *Haemonchus* spp. survived after LEV treatment, on farms B and C, LEV failed to control *Ostertagia* spp. (93% and 90% FECR, respectively). Remarkably, the use of IVM in combination with LEV achieved a 100% efficacy against all GI genera.

## 4. Discussion

The pharmaco-parasitological approach applied in this study constitutes a valuable tool for characterizing the relationship between pharmaceutical aspects, pharmacokinetic behavior, and therapeutic response, which is essential for optimizing parasite control in livestock. The main goal of the current work was to assess the pharmacokinetic and pharmacodynamic (drug effect) interactions after the combined use of IVM and LEV in cattle under kept natural field conditions. Such PK and PD assessments are essential to support the rational use and recommendation of drug combination to improve anthelmintic treatments.

Anthelmintic resistance in GI nematodes affecting livestock has become a global issue and is currently recognized as a major limitation to animal production. In Argentina, resistance to IVM was detected on 93% of cattle farms included in a nationwide survey [34]. Consistent with this finding, the analysis of the 90% confidence intervals for the three farms included in the present study confirmed the presence of IVM-resistant nematode populations, providing an appropriate scenario for the pharmacological evaluation of drug combinations as a scientifically relevant challenge. Notably, LEV was the only anthelmintic for which no resistance was reported in that survey [34], highlighting its preserved therapeutic efficacy under field conditions. In agreement with this observation, most of the nematode populations on farms A, B, and C were susceptible to LEV. In this context, nematodicidal drug combinations may represent a valuable strategy to delay the development of anthelmintic resistance and to control IVM-resistant parasite populations [4]. Indeed, modeling studies [35,36,37] indicate that the effectiveness of anthelmintic combinations largely depends on their implementation before resistance emerges to one or more of the active components.

When different anthelmintics are administered simultaneously, it is necessary to determine their disposition kinetics to understand any potential PK adverse interaction. Mean plasma concentration–time profiles obtained in the current study were similar to those reported for both IVM [13,38] and LEV [17] in previous studies in cattle. It is well established that the persistence of the broad-spectrum antiparasitic activity of IVM and other macrocyclic lactone endectocides relies on their slow disposition kinetics and pattern of plasma/tissues exchange in the host. The time of parasite exposure to active drug concentrations determines the efficacy and/or persistence of activity in ruminants [39,40]. As previously shown, IVM prepared in a non-aqueous formulation for SC injection is slowly absorbed to reach its plasma Cmax (30–36 ng/mL) at 3 days after administration, showing an extensive systemic exposure with a mean residence time of approximately 8 days (see Table 1). This pharmacokinetic behavior characterized for a good and slow absorption, extensive plasma/tissues exchange, low metabolism rate, large enterohepatic recycling and long persistence in the bloodstream (measured up to 30 days post-treatment) is well in agreement with its high lipophilicity. Although some metabolic products have been recovered in plasma after administration of IVM to cattle, this compound is minimally metabolized in cattle, bile and feces being the major routes of excretion for the unchanged parent drug [10]. In fact, in the present study, IVM parent drug was the main analyte recovered in plasma after SC administration of IVM to beef cattle. Additionally, IVM has been shown to be substrates of the P-gp transport protein, which participates in the mechanism of active biliary and intestinal secretion of different molecules from the bloodstream to the GI tract [10,41]. In fact, a significant increment in the systemic availability of IVM was obtained after its administration together with the antifungal drug itraconazole, a P-gp substrate [41]. However, all of these IVM kinetic features were not affected after its co-administration with LEV in the current study, which is relevant for the purpose of their combined use in cattle.

The information available on LEV PK in cattle is very scarce. Consistent with those earlier PK descriptions, SC administration of LEV (8 mg/kg) yielded a Cmax of 2.17 ± 0.76 µg/mL and an AUC_0–∞_ of 9.20 ± 2.77 µg·h/mL. LEV plasma concentrations decline over a period of 6 to 8 h, with 90% of the total dosage being excreted in 24 h. LEV is rapidly and extensively metabolized to a large number of metabolites in the liver. The main metabolizing pathways appear to be oxidation, hydrolysis and hydroxylation. Oxidation of the imidazothiazole ring is followed by oxidation to a carbonyl and hydrolysis to a thiohydantoic acid. Excretion of both LEV and metabolites (glucuronyl or S-cysteinyl-glycine conjugates) is mainly in the urine (about 60%) and feces (about 30%) [10]. Although the relatively short persistence of LEV (T½el 6.22 ± 1.08 h) would not reduce the selective pressure exerted by the longer-acting component during the terminal phase of the IVM elimination curve (T½el 4.62 ± 0.95 days), this situation is comparable to that observed when IVM is administered alone [6]. Therefore, the initial overlap between the time-to-kill profiles of IVM and LEV (at the early stages post-treatment) following their co-administration is critical to achieve simultaneous “lethal” systemic exposure, thereby maximizing the pharmacodynamic effect and overall therapeutic efficacy.

The concurrent administration of two drug compounds may result in pharmacokinetic (PK) interactions that alter the systemic exposure of one or both agents. Therefore, the evaluation of potential PK interferences is essential when combination therapies are considered. In the present study, no adverse PK interactions were observed following the combined SC administration of IVM and LEV in calves. Comparative analysis revealed no statistically significant differences in any of the evaluated PK parameters between the single-drug and combination treatments (Table 1). In addition, the plasma concentration–time profiles were essentially superimposable under both treatment conditions (Figure 2 and Figure 3), indicating the absence of clinically relevant PK interactions.

PK interactions among anthelmintic drugs have been more extensively characterized in sheep. For example, Alvarez [42] demonstrated that co-administration of albendazole and IVM in lambs resulted in altered systemic exposure, indicating a clear PK interaction. Similarly, Suarez [43] reported drug–drug interactions following the combined administration of IVM, albendazole, and LEV. In contrast with these findings in sheep, the present study in cattle did not reveal significant PK alterations for either IVM or LEV after their SC co-administration, suggesting species differences in interaction profiles. Although PK interactions among nematodicidal drugs have been less extensively investigated in cattle, available evidence supports a limited or compound-specific interaction pattern. Leathwick [44] observed increased systemic availability of abamectin when administered orally in combination with LEV, while no changes were detected in LEV plasma profiles under the same conditions, indicating that interactions may not affect all compounds equally. Similarly, Cromie [45] reported no differences in the plasma PK profiles of IVM and closantel administered subcutaneously to cattle, either alone or as a combined formulation. Moreover, no PK interactions were observed after the combined SC administration of LEV and RBZ in calves [17]. Overall, the results obtained in the present study are consistent with previous reports in cattle, supporting the conclusion that the PK of each active compound are not significantly influenced by the presence of a co-administered anthelmintic.

The independence of the molecular targets of IVM and LEV (different modes of antiparasitic action) supports the rationale behind the proposed combined administration in cattle under assessment in the current work. GI parasitism in cattle commonly involves multiple parasite genera with varying susceptibility profiles. The co-administration of distinct drugs with different mechanisms of action may improve overall efficacy by ensuring that parasites surviving exposure to one compound are effectively targeted by the other, thereby optimizing systemic exposure and enhancing the pharmacodynamic response of the combination. An IVM failure to control the GI nematodes *Cooperia* spp. and *Haemonchus* spp. was observed, which is consistent with previous reports [2,34,38,46]. Since *Cooperia* spp. is a dose-limiting species for IVM, this is the nematode genus in which IVM resistance would be first expected [47]. Although LEV alone achieved high overall therapeutic activities, it did not show effective control against all the GI nematodes present in the calves on farms B and C. Indeed, on these farms, LEV offered only a limited control in *Ostertagia* spp. (93% and 90% FECR, respectively). These findings are also consistent with those from a field trial in the United States, in which the overall efficacy of LEV, against all stages of *Ostertagia ostertagi*, was consistently low [48]. A similar reduced efficacy of LEV has been reported in other countries (i.e., New Zealand) against different GI nematodes [49]. Remarkably, the experimental use of IVM in combination with LEV in the current trial achieved 100% efficacy, with maximum therapeutic activity (pharmacodynamic assessment) against all GI parasite genera, supporting the rationale for using this nematodicidal combination. Notably, the assayed combination was the only treatment that achieved a full therapeutic response (100% clinical efficacy) on all the farms. This outcome is in line with the expected additive synergic activity between the two molecules [7], whereby the combined effect corresponds to the sum of the individual drug effects [50].

The detrimental effects of inadequate control of resistant GI nematodes on cattle productivity have been well established [51,52,53]. This negative impact was observed on farm A in the trial described here, where the mean weight gain after 44 days was 0.6 kg (IVM alone), 5.9 (LEV alone) and reached up to 8.4 kg for the combined IVM + LEV treatment, reflecting the better performance in parasite control and weight gain after administration of the concurrent treatment. If LEV still retains high efficacy, their combined use may serve as a valuable pharmacological strategy to delay the development of resistance. Ideally, when an anthelmintic treatment achieves 100% efficacy (as observed in the present study), selection for resistant parasites is effectively prevented [54]. Based on the described PK and PD assessments, the combination of a long-acting drug (IVM) with a short-acting compound (LEV), appears to be a promising pharmacological option for controlling resistant GI nematodes in cattle, with the additional potential to delay the progression of nematode anthelmintic resistance.

Some limitations in the planning and execution of the present study should be acknowledged. The interpretation of the data could have been strengthened by refinements in the experimental design, particularly to better elucidate the interaction (pharmacodynamics) between both molecules at their sites of antiparasitic action. This would require measuring concentrations of both molecules within the target parasites and conducting interaction analyses to characterize the nature of the synergistic activity underlying the observed antiparasitic response. Despite these limitations, the present work provides original and valuable data on the integrated pharmaco-therapeutic characterization of the combined administration of two anthelmintic molecules. Overall, the work presented here contributes sound pharmacology data highly useful to optimize parasite control in livestock. The described drug combination supported with original scientific data, may contribute to enhancing the antiparasitic therapeutic outcome, while promoting more sustainable parasite management practices in cattle production systems.

## Figures and Tables

**Table 1:** Plasma pharmacokinetic parameters (mean ± SD) of ivermectin (IVM) following subcutaneous administration (0.2 mg/kg), either as a single treatment or co-administered with levamisole (LEV, 8 mg/kg), in naturally parasitized calves.

IVERMECTIN			
Pharmacokinetic Parameters	IVM Alone	IVM + LEV	*p* Value
T_max_ (d)	3.40 ± 2.19	3.00 ± 0.00	0.5275
C_max_ (ng/mL)	32.9 ± 21.7	36.0 ± 16.7	0.7859
AUC_0–t_ (ng.d/mL)	274 ± 65.1	295 ± 111	0.7188
AUC_0–∞_ (ng.d/mL)	278 ± 64.5	300 ± 110	0.7056
MRT (d)	8.08 ± 1.95	7.96 ± 2.54	0.9298
T_½el_ (d)	4.62 ± 0.95	4.35 ± 2.21	0.8077

T_max_: time to peak plasma concentration; C_max_: peak plasma concentration; AUC_0–t_: area under the plasma concentration vs. time curve from 0 to time; AUC_0–__∞_: area under the concentration–time curve extrapolated to infinity; MRT: mean residence time; T_½el_: elimination half-life. For all pharmacokinetic parameters, *p* > 0.05.

**Table 2:** Plasma pharmacokinetic parameters (mean ± SD) for levamisole (LEV) obtained after its subcutaneous administration (8 mg/kg) both alone and co-administered with ivermectin (IVM) (0.2 mg/kg) to naturally parasitized calves.

LEVAMISOLE			
Pharmacokinetic Parameters	LEV Alone	LEV + IVM	*p* Value
T_max_ (h)	1.14 ± 0.38	1.00 ± 0.00	>0.999
C_max_ (µg/mL)	2.17 ± 0.76	1.91 ± 0.57	0.4930
AUC_0–t_ (µg.h/mL)	8.90 ± 2.69	9.11 ± 1.82	0.8701
AUC_0–∞_ (µg.h/mL)	9.20 ± 2.77	9.40 ± 1.97	0.9134
MRT (h)	6.50 ± 1.59	6.10 ± 0.99	0.6223
T_½el_ (h)	6.22 ± 1.08	5.41 ± 0.48	0.0937

T_max_: time to peak plasma concentration; C_max_: peak plasma concentration; AUC_0–t_: area under the plasma concentration vs. time curve from 0 to time; AUC_0–__∞_: area under the concentration–time curve extrapolated to infinity; MRT: mean residence time; T_½el_: elimination half-life. For all pharmacokinetic parameters, *p* > 0.05.

**Table 3:** Nematode egg per gram counts (EPG, arithmetic mean, range), therapeutic response expressed as the reduction percentages of fecal egg counts (FECR) (undifferentiated), lower and upper confidence intervals 90%, and nematode population status after the subcutaneous administration of ivermectin (IVM, 0.2 mg/kg) and levamisole (LEV, 8 mg/kg) given both separately and co-administered to naturally parasitized calves.

Farm ID	Experimental Group (*n* = 15)	EPG Counts (Range)		Therapeutic Response FECRT ^1^	90% CI ^2^	Nematode Population Status
		Day 0	Day 14			
FARM A	IVM Alone	657 ^a^ (340–1400)	403 ^a^ (40–1120)	38.7%	13.8–60.0%	Resistant
	LEV Alone	637 ^a^ (280–1300)	1.30 ^b^ (0–20)	99.6%	99.4–100%	Susceptible
	Combination IVM + LEV	796 ^a^ (320–1280)	0.00 ^b^ (0–0)	100%	-	Susceptible
	IVM Alone	469 ^a^ (100–1460)	269 ^a^ (0–1060)	42.6%	3.30–72.8%	Resistant
FARM B	LEV Alone	559 ^a^ (180–1260)	2.20 ^b^ (0–20)	99.6%	99.1–99.9%	Susceptible
	Combination IVM + LEV	569 ^b^ (100–1820)	0.00 ^b^ (0–0)	100%	-	Susceptible
FARM C	IVM Alone	437 ^a^ (200–980)	217 ^a^ (0–580)	54%	3.70–83.0%	Resistant
	LEV Alone	309 ^a^ (260–1140)	2.90 ^b^ (0–20)	99.1%	96.8–100%	Susceptible
	Combination IVM + LEV	266 ^a^ (240–760)	0.00 ^b^ (0–0)	100%	-	Susceptible

^1^ FECRT estimated according to [33]. ^2^ 90% CI: lower and upper confidence intervals estimated according to [31]. EPG counts with different superscript letters are statistically different (*p* < 0.05).

**Table 4:** Therapeutic response measured as the reduction percentages of fecal egg counts (FECRT) for *Cooperia*, *Haemonchus*, *Ostertagia* and *Oesophagostomum* spp. (based on egg counts partitioned to genera using the proportion of each genus recovered as larvae from fecal larval cultures) after the subcutaneous administration of ivermectin (IVM, 0.2 mg/kg) and levamisole (LEV, 8 mg/kg) given both separately and co-administered to naturally parasitized calves.

Farm ID	Experimental Group	FECRT Day 14 ^1^			
		*Cooperia*	*Haemonchus*	*Ostertagia*	*Oesophagostomum*
FARM A	IVM Alone	35.1%	34.7%	100%	100%
	LEV Alone	100%	99.6%	100%	100%
	Combination IVM + LEV	100%	100%	100%	100%
	IVM Alone	87.2%	8.60%	100%	100%
FARM B	LEV Alone	100%	100%	93.4%	100%
	Combination IVM + LEV	100%	100%	100%	100%
FARM C	IVM Alone	40.9%	100%	100%	100%
	LEV Alone	100%	100%	90.7%	100%
	Combination IVM + LEV	100%	100%	100%	100%

^1^ FECR estimated according to [33].

## Data Availability

The original contributions presented in this study are included in the article. Further inquiries can be directed to the corresponding author.
